# miR-100 Induces Epithelial-Mesenchymal Transition but Suppresses Tumorigenesis, Migration and Invasion

**DOI:** 10.1371/journal.pgen.1004177

**Published:** 2014-02-27

**Authors:** Dahu Chen, Yutong Sun, Yuan Yuan, Zhenbo Han, Peijing Zhang, Jinsong Zhang, M. James You, Julie Teruya-Feldstein, Min Wang, Sumeet Gupta, Mien-Chie Hung, Han Liang, Li Ma

**Affiliations:** 1 Department of Experimental Radiation Oncology, The University of Texas MD Anderson Cancer Center, Houston, Texas, United States of America; 2 Department of Molecular and Cellular Oncology, The University of Texas MD Anderson Cancer Center, Houston, Texas, United States of America; 3 Department of Bioinformatics and Computational Biology, The University of Texas MD Anderson Cancer Center, Houston, Texas, United States of America; 4 Graduate Program in Structural and Computational Biology and Molecular Biophysics, Baylor College of Medicine, Houston, Texas, United States of America; 5 Department of Hematopathology, The University of Texas MD Anderson Cancer Center, Houston, Texas, United States of America; 6 Cancer Biology Program, Graduate School of Biomedical Sciences, The University of Texas Health, Science Center at Houston, Houston, Texas, United States of America; 7 Department of Pathology, Memorial Sloan-Kettering Cancer Center, New York, New York, United States of America; 8 Whitehead Institute for Biomedical Research, Cambridge, Massachusetts, United States of America; Cincinnati Children's Hospital Medical Center, United States of America

## Abstract

Whether epithelial-mesenchymal transition (EMT) is always linked to increased tumorigenicity is controversial. Through microRNA (miRNA) expression profiling of mammary epithelial cells overexpressing Twist, Snail or ZEB1, we identified miR-100 as a novel EMT inducer. Surprisingly, miR-100 inhibits the tumorigenicity, motility and invasiveness of mammary tumor cells, and is commonly downregulated in human breast cancer due to hypermethylation of its host gene *MIR100HG*. The EMT-inducing and tumor-suppressing effects of miR-100 are mediated by distinct targets. While miR-100 downregulates E-cadherin by targeting *SMARCA5*, a regulator of *CDH1* promoter methylation, this miRNA suppresses tumorigenesis, cell movement and invasion *in vitro* and *in vivo* through direct targeting of *HOXA1*, a gene that is both oncogenic and pro-invasive, leading to repression of multiple HOXA1 downstream targets involved in oncogenesis and invasiveness. These findings provide a proof-of-principle that EMT and tumorigenicity are not always associated and that certain EMT inducers can inhibit tumorigenesis, migration and invasion.

## Author Summary

Induction of epithelial-mesenchymal transition (EMT) in epithelial tumor cells has been shown to enhance migration, invasion and cancer ‘stemness’. Here we demonstrate that a miRNA downregulated in human breast tumors, miR-100, can simultaneously induce EMT and inhibit tumorigenesis, migration and invasion through direct targeting of distinct genes. This is the first report of an EMT inducer that suppresses cell movement and tumor invasion, which indicates that EMT is not always associated with increased tumorigenesis, migration and invasion, and that all EMT inducers are not equal: while some of them can promote tumorigenicity, motility and invasiveness, others inhibit these properties owing to their ability to concurrently target both EMT-repressing genes and oncogenic/pro-invasive genes. These findings provide new insights into the complex roles of EMT inducers.

## Introduction

Epithelial-mesenchymal transition (EMT) is regulated by transcription factors [1], [2], extracellular ligands [3] and microRNAs (miRNAs) [4]–[9]. It has been proposed that inducing EMT in epithelial tumor cells enhances migration, invasion and dissemination, whereas the mesenchymal-epithelial transition (MET) process facilitates metastatic colonization [1], [2], [10]–[12]. In addition, induction of EMT in differentiated tumor cells has been shown to generate cells with properties of tumor-initiating cells, or cancer stem cells [13], [14]. However, whether EMT and tumorigenicity are always linked is debated. Recently, analysis of clonal populations derived from the PC-3 prostate cancer cell line demonstrated that a metastatic clone was highly proliferative and expressed genes associated with an epithelial phenotype, whereas a non-metastatic clone was poorly proliferative and expressed genes associated with EMT [15]. Whether this finding is attributed to clonal bias or holds true in general is unknown. Moreover, whether there exist specific gene products that concurrently induce EMT and inhibit tumorigenesis remains elusive.

## Results

### miR-100 induces epithelial-mesenchymal transition

To systematically identify miRNAs differentially expressed in EMT, we overexpressed EMT-inducing transcription factors, Twist, Snail or ZEB1, in the experimentally immortalized, non-transformed human mammary epithelial cells [16], termed HMLE cells. Each of these transcription factors was capable of inducing EMT, as evidenced by changes in morphology (**Figure S1A**), downregulation of E-cadherin (*CDH1*), and upregulation of N-cadherin (*CDH2*), vimentin (*VIM*) and multiple EMT-inducing transcription factors (**Figure S1B**).

**Figure 1:**
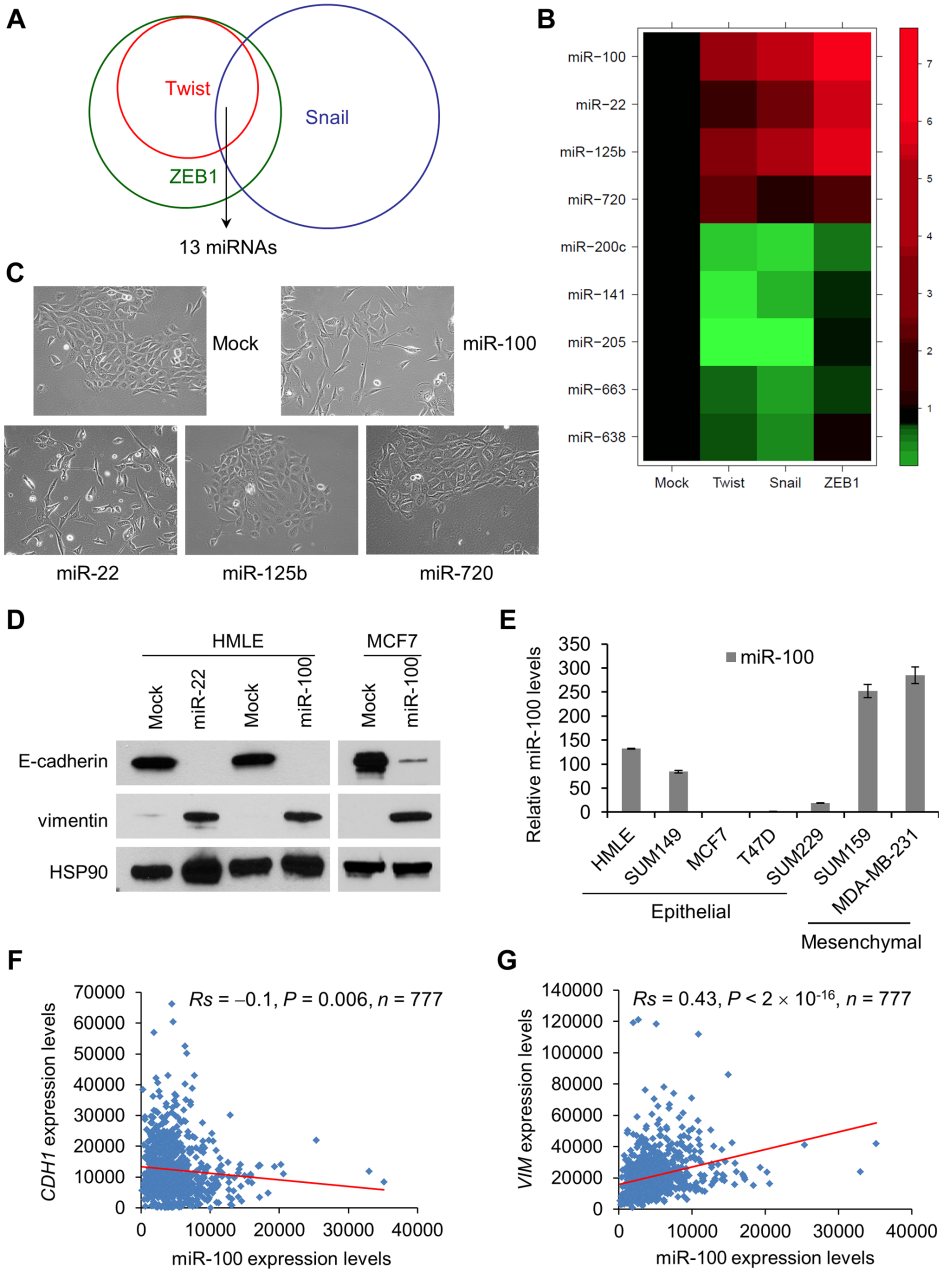
miR-100 induces EMT and correlates with the EMT state in human breast cancer. (A) Venn diagram representation of the 13 miRNAs that are commonly deregulated in HMLE cells transduced with Twist, Snail or ZEB1, compared with mock-infected HMLE cells. (B) Heat map showing expression levels of the nine miRNAs validated by TaqMan qPCR. (C) Phase contrast images of HMLE cells transduced with miR-100, miR-22, miR-125b or miR-720. (D) Immunoblotting of E-cadherin, vimentin and HSP90 in HMLE cells transduced with miR-100 or miR-22, and in MCF7 cells transduced with miR-100. (E) qPCR of miR-100 in a series of human breast cancer cell lines. Data are mean ± SEM. (F) Correlation of miR-100 with *CDH1* (F) and *VIM* (G) expression levels in clinical breast cancer, based on the RNA-Seq data from TCGA. Statistical significance was determined by Spearman rank correlation test. *Rs* = Spearman rank correlation coefficient.

Next, we performed miRNA microarray profiling analysis (**Table S1**) of these HMLE cells that had been induced to undergo EMT and identified a set of 13 EMT-associated miRNAs (**Figure 1A** **and S2A**; **Table S2**). Using TaqMan qPCR assays, we confirmed that four miRNAs, miR-100, miR-125b, miR-22 and miR-720, were commonly upregulated miRNAs in EMT; five miRNAs, miR-200c, miR-141, miR-205, miR-663 and miR-638, were commonly downregulated miRNAs in EMT (**Figure 1B** and **Table S2**). The most dramatically deregulated miRNAs were miR-205 and two clustered miR-200 family members – miR-200c and miR-141 (**Table S2**), which are the first EMT-regulating miRNAs discovered through other approaches [4], [5]. This result validated the efficacy of our experimental system.

Differentially expressed miRNAs could be either causes or consequences of EMT. We cloned the four upregulated miRNAs into puromycin resistance cassette-containing retroviral vectors (MSCV-PIG and pBabe-puro) and expressed them individually in HMLE cells. While miR-125b and miR-720 did not cause any changes in cell morphology or EMT markers (**Figure 1C** and data not shown), expression of either miR-100 or miR-22 (**Figure S2B**) was sufficient to induce EMT: upon expression of either miRNA, epithelial cells became scattered and assumed fibroblastic morphology (**Figure 1C**); E-cadherin expression was undetectable and the mesenchymal marker vimentin was dramatically induced (**Figure 1D**). Similarly, expression of miR-100 in the MCF7 human epithelial breast cancer cell line (**Figure S2C**) also markedly downregulated E-cadherin and upregulated vimentin (**Figure 1D**), although we did not observe a clear morphological change.

We examined miR-100 expression levels in a series of human breast cancer cell lines. Relative to HMLE cells, epithelial-like tumor cell lines exhibited either comparable or much lower miR-100 expression, whereas mesenchymal-like tumor cell lines showed higher levels of miR-100 (**Figure 1E**). The association between miR-100 and EMT markers was further validated in human tumors: from the Cancer Genome Atlas (TCGA) breast cancer data [17], we observed a moderate but significant inverse correlation between miR-100 and E-cadherin expression levels (*Rs* = −0.1, *P* = 0.006, **Figure 1F**) and a highly significant positive correlation between miR-100 and vimentin expression levels (*Rs* = 0.43, *P*<2×10^−16^, **Figure 1G**).

### miR-100 suppresses tumor growth and is downregulated in human breast cancer

**Figure 2:**
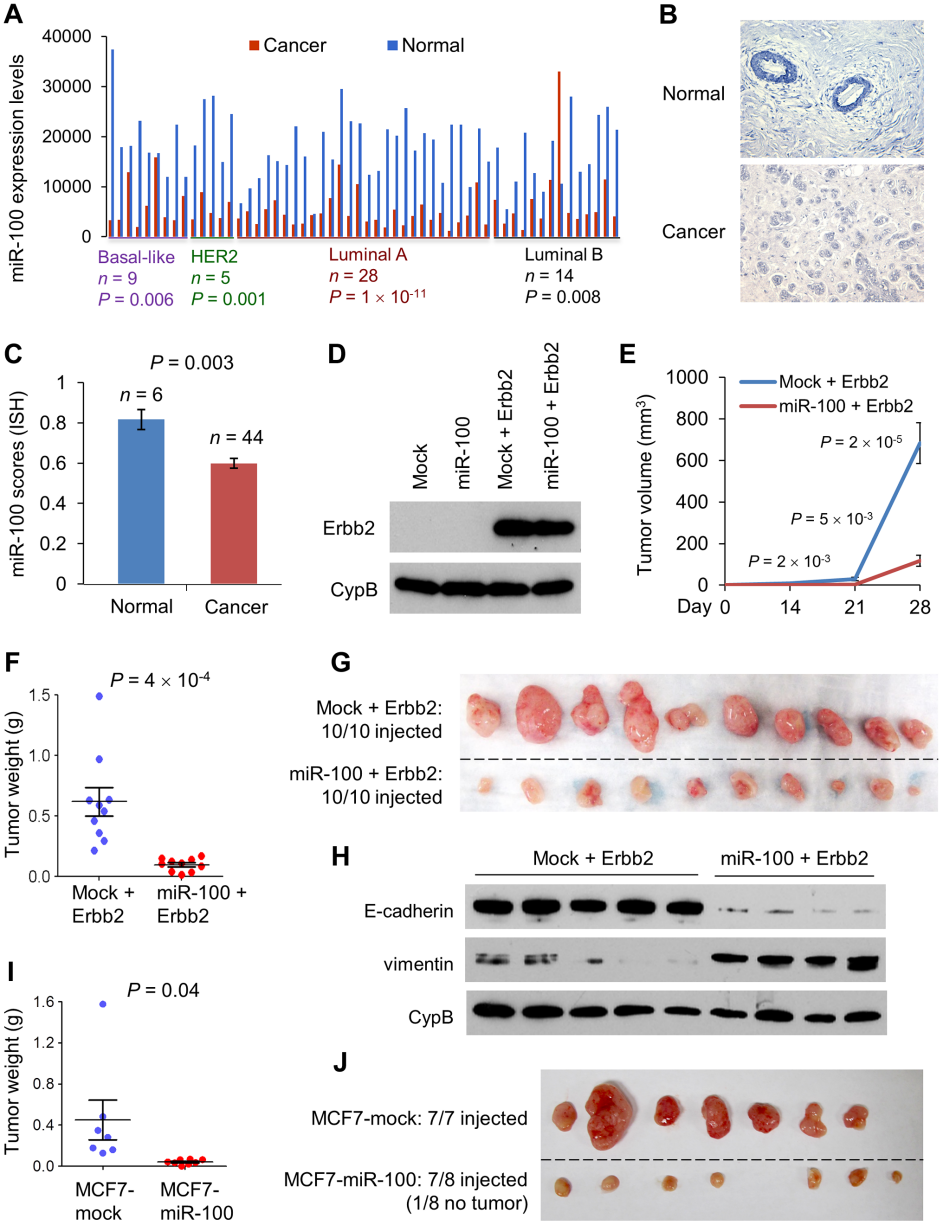
miR-100 inhibits tumorigenesis and is downregulated in human breast cancer. (A) miR-100 expression levels in four subtypes of human breast tumors and paired normal breast tissues, based on the RNA-Seq data from TCGA. Statistical significance was determined by paired *t* test. (B) Representative images of *in situ* hybridization of miR-100 in normal human mammary glands and human breast carcinomas. Blue color indicates positive staining. (C) miR-100 scores (normalized to U6) based on *in situ* hybridization of human breast tissue microarray (TMA). (D) Immunoblotting of Erbb2 and cyclophilin B (CypB) in HMLE cells transduced with miR-100 and Erbb2, alone or in combination. (E) Tumor growth by 1.5×10^6^ subcutaneously injected HMLE cells transduced with Erbb2 alone or in combination with miR-100. Data are mean ± SEM (*n* = 10 mice per group). (F, G) Tumor weight (F) and tumor images (G) of mice with subcutaneous injection of HMLE cells transduced with Erbb2 alone or in combination with miR-100, at day 31 after implantation. Data in (F) are mean ± SEM (*n* = 10 mice per group). (H) Immunoblotting of E-cadherin, vimentin and cyclophilin B (CypB) in tumor lysates from (G). (I, J) Tumor weight (I) and tumor images (J) of mice with subcutaneous injection of 5×10^6^ miR-100-transduced MCF7 cells, at day 22 after implantation. Data in (I) are mean ± SEM (*n* = 7–8 mice per group). Statistical significance in (C), (E), (F) and (I) was determined by two-tailed, unpaired Student's *t* test.

We performed TCGA data analysis to determine the expression levels of miR-100 and miR-22 in human breast cancer. Surprisingly, miR-100 was found to be downregulated in all subtypes of human breast tumors, including luminal A (*P* = 1×10^−11^), luminal B (*P* = 0.008), basal-like (*P* = 0.006) and HER2 (*P* = 0.001) subtypes, compared with paired normal breast tissues (**Figure 2A**). Consistent with the correlation of miR-100 with EMT markers (**Figure 1F and 1G**), the luminal A subtype of primary breast tumors (which are known to be E-cadherin-positive and vimentin-negative) exhibited the most significant downregulation of miR-100 (**Figure 2A**). In contrast, miR-22 expression showed no significant difference between cancer and paired normal tissues (**Figure S3**). To determine the cellular origin of miR-100 expression, we performed *in situ* hybridization on human normal and cancer tissues, and found that miR-100 was indeed highly expressed in normal human mammary epithelium as opposed to barely detectable expression in the stroma, whereas human breast tumors exhibited reduced miR-100 expression (**Figure 2B and 2C**). Therefore, downregulation of miR-100 reflects the difference between normal mammary epithelium and breast tumor cells, but is not due to the difference in the stroma.

This observed downregulation of miR-100 in human breast tumors prompted us to determine whether it could be a tumor suppressor. Indeed, expression of miR-100 significantly inhibited the proliferation of HMLE cells *in vitro*, either in the presence or absence of ectopic expression of the *Erbb2* mammary oncogene (**Figure 2D** **and S4A**). To validate this effect *in vivo*, we subcutaneously implanted Erbb2-expressing HMLE cells (HMLE-Erbb2) with or without miR-100 overexpression into nude mice. Strikingly, miR-100 expression dramatically suppressed tumor formation and growth (**Figure 2E–2G**), as it not only delayed initial tumor onset by one week (**Figure 2E**), but also caused a 83% reduction in tumor volume (683.3 mm^3^ vs. 117.2 mm^3^, **Figure 2E**) and a 84% reduction in tumor weight (0.62 g vs. 0.098 g, **Figure 2F and 2G**) at the late stage. Western blot analysis of E-cadherin and vimentin in tumor lysates (**Figure 2H**) and E-cadherin immunohistochemical staining of the tumors (**Figure S4B**) confirmed that the EMT status was retained in tumors formed by miR-100-expressing HMLE-Erbb2 cells. Furthermore, a 91% decrease in tumor weight was observed in mice implanted with miR-100-overexpressing MCF7 human breast cancer cells, compared with hosts of mock-infected MCF7 cells (**Figure 2I** and **2J**).

### miR-100 regulates EMT and tumorigenesis by targeting distinct genes

**Figure 3:**
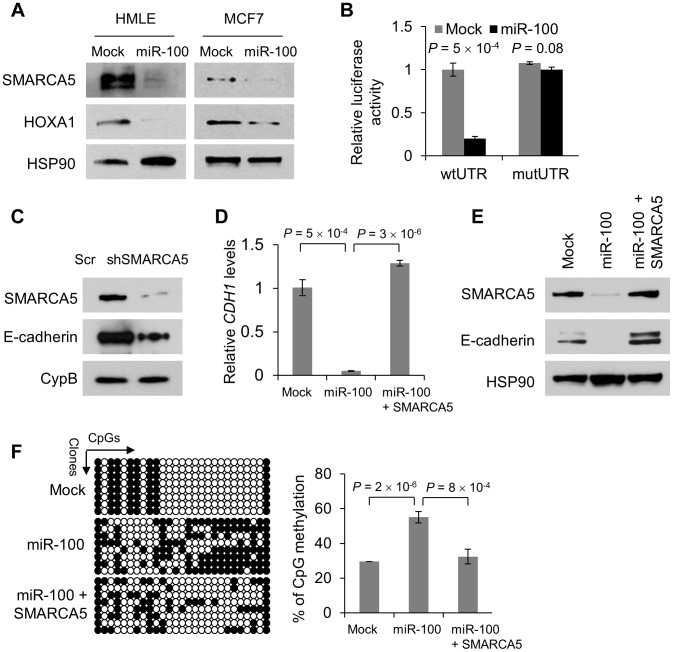
miR-100 downregulates E-cadherin by targeting *SMARCA5*. (A) Immunoblotting of SMARCA5, HOXA1 and HSP90 in HMLE and MCF7 cells transduced with miR-100. (B) Luciferase activity of the wild-type or mutant *HOXA1* 3′ UTR reporter gene in 293T cells with ectopic expression of miR-100. (C) Immunoblotting of SMARCA5, E-cadherin and cyclophilin B (CypB) in HMLE cells infected with the SMARCA5 shRNA (shSMARCA5) or the pLKO.1-puro lentiviral vector with a scrambled sequence (Scr) that does not target any mRNA. (D) qPCR of *CDH1* in HMLE cells transduced with the control vector (mock), miR-100 alone or in combination with SMARCA5. (E) Immunoblotting of SMARCA5, E-cadherin and HSP90 in HMLE cells transduced with the control vector (mock), miR-100 alone or in combination with SMARCA5. (F) Bisulfite sequencing assay (left panel) and the percentage of CpG methylation (right panel) of the *CDH1* promoter in HMLE cells transduced with the control vector (mock), miR-100 alone or in combination with SMARCA5. Open circles: unmethylated CpG sites; solid black circles: methylated CpG sites. Data in (B), (D) and (F) are mean ± SEM, and statistical significance was determined by two-tailed, unpaired Student's *t* test.

We hypothesized that different target genes of miR-100 mediate the two distinct functions of this miRNA. Four miR-100 targets, *SMARCA5*, *SMARCD1*, *MTOR* (mammalian target of rapamycin) and *BMPR2*, have been identified by reporter assays previously [18], [19]. In addition, among all predicted targets of miR-100, *HOXA1* is a mammary oncogene [20] and is upregulated in human breast cancer [21]; overexpression of HOXA1 in immortalized human mammary epithelial cells was sufficient to induce aggressive tumor formation *in vivo* [20]. While miR-100 did not substantially alter expression levels of SMARCD1, mTOR and BMPR2 in HMLE cells (**Figure S5A**), overexpression of this miRNA in both HMLE and MCF7 cells resulted in a pronounced decrease in SMARCA5 and HOXA1 protein levels (**Figure 3A**). Moreover, the activity of a luciferase reporter fused to a wild-type *HOXA1* 3′ UTR, but not that of a reporter fused to a mutant *HOXA1* 3′ UTR with mutations in the miR-100 binding site (**Figure S5B**), was reduced by 80% upon expression of miR-100 (**Figure 3B**), which validated *HOXA1* as a direct target of this miRNA.

We silenced *SMARCA5* in HMLE cells. This markedly reduced E-cadherin protein expression (**Figure 3C**) but did not alter cell proliferation (**Figure S5C**), suggesting that downregulation of SMARCA5 partially mediates the EMT-inducing effect of miR-100 but not its growth-inhibitory function. Conversely, re-expression of SMARCA5 in miR-100-overexpressing HMLE cells restored the expression of E-cadherin at both mRNA and protein levels (**Figure 3D** and **3E**), although the mesenchymal morphology was not reversed. SMARCA5 (also named hSNF2H) is a chromatin-remodeling protein that physically interacts with the DNA methyltransferase DNMT3B [22]. Although it is not clear how this interaction modulates DNMT3B activity, we speculated that miR-100 might promote *CDH1* (encoding E-cadherin) gene methylation by targeting *SMARCA5*. Indeed, bisulfite sequencing assays of the 27 CpG sites in the *CDH1* promoter region revealed 29.6% methylation in the control HMLE cells and 55.1% methylation in miR-100-overexpressing HMLE cells, while re-expression of SMARCA5 reversed the effect of miR-100 on *CDH1* promoter methylation (**Figure 3F**).

**Figure 4:**
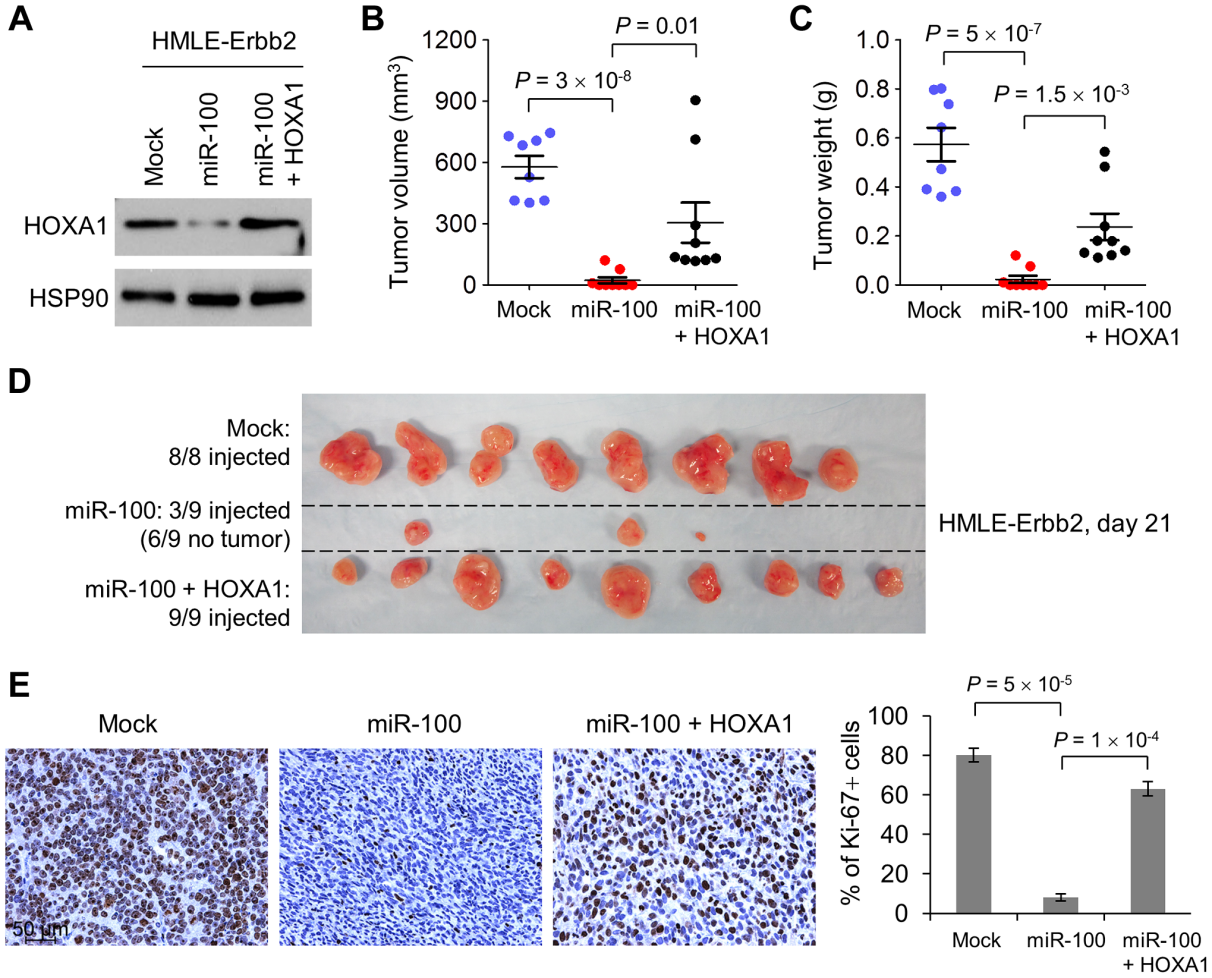
miR-100 suppresses tumorigenesis by targeting *HOXA1*. (A) Immunoblotting of HOXA1 and HSP90 in Erbb2-expressing HMLE cells (HMLE-Erbb2) transduced with the control vector (mock), miR-100 alone or in combination with HOXA1. (B–D) Tumor volume (B), tumor weight (C) and tumor images (D) of mice with subcutaneous injection of 3×10^6^ HMLE-Erbb2 cells transduced with the control vector (mock), miR-100 alone or in combination with HOXA1, at day 21 after implantation. Data in (B) and (C) are mean ± SEM (*n* = 8–9 mice per group). (E) Ki-67 immunohistochemical staining (left panel) and the percentage of Ki-67-positive cells (right panel) in the tumors formed by HMLE-Erbb2 cells transduced with the control vector (mock), miR-100 alone or in combination with HOXA1, at day 21 after implantation. Scale bar: 50 µm. Data are mean ± SEM (*n* = 3 mice per group). Statistical significance in (B), (C) and (E) was determined by two-tailed, unpaired Student's *t* test.

In contrast to the effect of SMARCA5, restoring HOXA1 expression in miR-100-overexpressing HMLE-Erbb2 cells to the same level as the control HMLE-Erbb2 cells (**Figure 4A**) did not affect expression levels of EMT-associated markers (**Figure S5D**), but instead fully rescued tumor onset and partially rescued tumor volume (51% rescue, **Figure 4B**) and tumor weight (40% rescue, **Figure 4C** and **4D**). Consistent with the *in vitro* effect of miR-100 on EMT induction (**Figure 1C** and **1D**) and cell proliferation (**Figure S4A**), the control HMLE-Erbb2 tumors were epithelial and had 80% Ki-67-positive cells, miR-100-expressing HMLE-Erbb2 tumors exhibited mesenchymal morphology and 8% Ki-67-positive cells, whereas HMLE-Erbb2 tumors with co-expression of miR-100 and HOXA1 were mesenchymal but showed 63% Ki-67-positive cells (**Figure 4E**). Taken together, downregulation of HOXA1 mediates, at least in part, the tumor-suppressing effect of miR-100 but not its EMT-inducing function.

### miR-100 inhibits migration and invasion by targeting *HOXA1*

**Figure 5:**
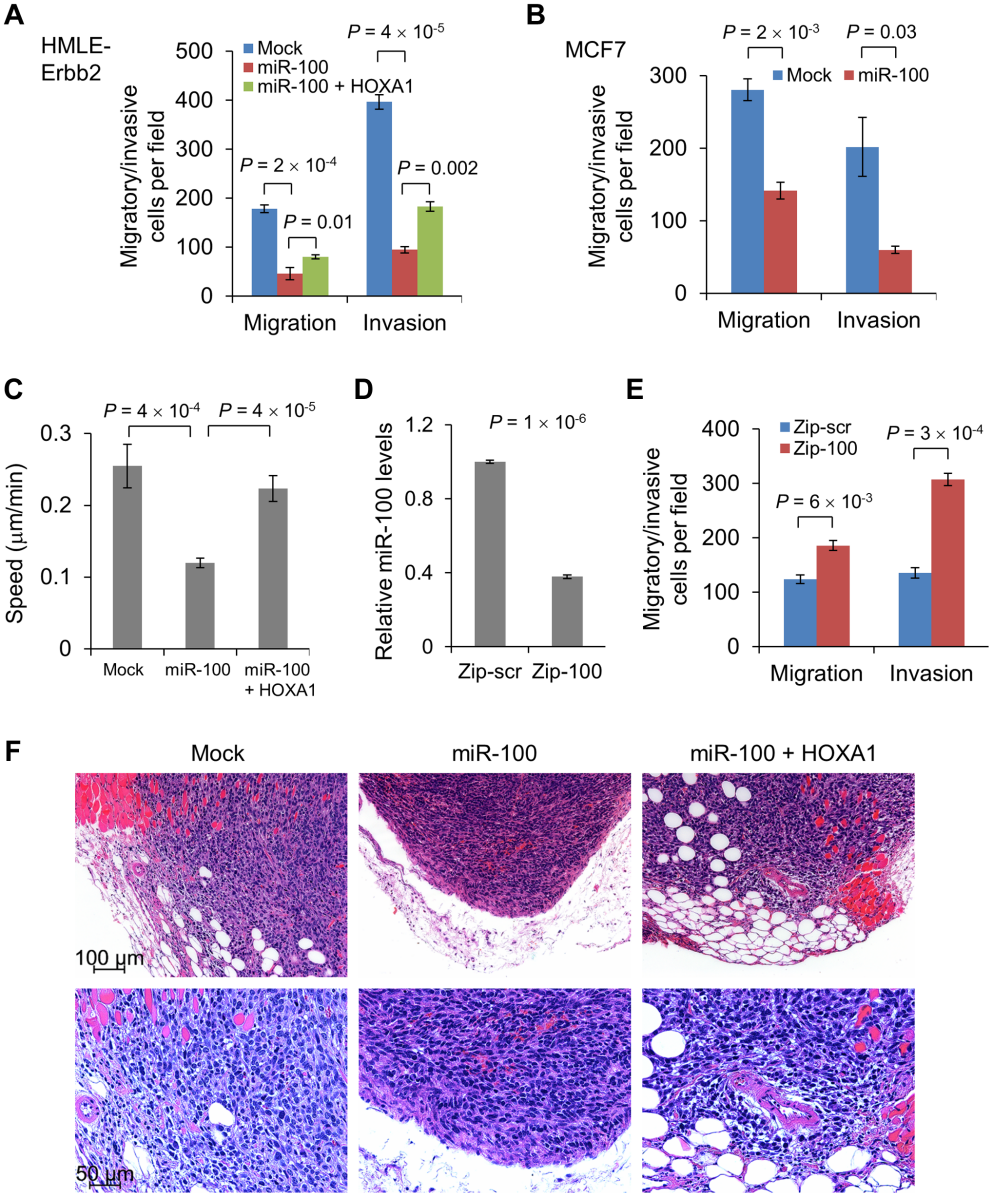
miR-100 inhibits migration and invasion by targeting *HOXA1*. (A) Transwell migration and Matrigel invasion assays of HMLE-Erbb2 cells transduced with the control vector (mock), miR-100 alone or in combination with HOXA1. (B) Transwell migration and Matrigel invasion assays of miR-100-transduced MCF7 cells. (C) Quantification of the speed of movement (µm/min, *n* = 10 cells per group) of HMLE-Erbb2 cells transduced with the control vector (mock), miR-100 alone or in combination with HOXA1. (D) qPCR of miR-100 in MDA-MB-231 cells expressing a short hairpin inhibiting miR-100 (Zip-100) or a scrambled hairpin control (Zip-scr). (E) Transwell migration and Matrigel invasion assays of MDA-MB-231 cells expressing a short hairpin inhibiting miR-100 (Zip-100) or a scrambled hairpin control (Zip-scr). Data in (A)–(E) are mean ± SEM, and statistical significance was determined by two-tailed, unpaired Student's *t* test. (F) H & E staining of the tumors formed by HMLE-Erbb2 cells transduced with the control vector (mock), miR-100 alone or in combination with HOXA1, at day 21 after implantation. Scale bar: 100 µm in upper panels and 50 µm in lower panels.

Unexpectedly, despite strong EMT induction in both HMLE-Erbb2 and MCF7 cells, expression of miR-100 suppressed their migration and invasion *in vitro*, as gauged by Transwell assays (**Figure 5A** and **5B**; **Figure S6A** and **S6B**). To further confirm the inhibitory effect of miR-100 on cell motility, we tracked the movement of individual cells cultured on top of collagen over a 24-hour period. Using time-lapse video microscopy, we observed a 53% decrease in the speed of movement of miR-100-expressing HMLE-Erbb2 cells compared with HMLE-Erbb2 cells (**Figure 5C**; **Video S1** and **S2**). It should be noted that in order to permit the space for cell movement, the condition used for this experiment was low density and did not allow the majority of HMLE-Erbb2 cells to form epithelial clusters; however, we did observe HMLE-Erbb2 cell clusters with epithelial island structure that exhibited a surprisingly rapid collective movement and long trajectories without cell dissociation (**Video S1** – note that an epithelial cell cluster initially appeared in the upper left corner and then moved to the lower part of the field), whereas all miR-100-expressing HMLE-Erbb2 cells had highly limited area of movement and reduced speed (**Video S2**).

To our knowledge, this is the first time that conversion from an epithelial state to a mesenchymal state has been found to be accompanied by reduced motility and invasiveness, which indicates that miR-100 may concurrently target EMT-repressing genes (*SMARCA5*) and pro-invasive genes. Indeed, *HOXA1* has been identified as a driver of both oncogenesis and the invasion-metastasis cascade in human melanoma [23]. Consistent with this finding, restoration of HOXA1 in miR-100-overexpressing HMLE-Erbb2 cells (**Figure 4A**) rescued cell migration and invasion (**Figure 5A** and **5C**; **Figure S6A**; **Video S3**). In contrast, neither re-expression of SMARCA5 in miR-100-overexpressing HMLE cells nor knockdown of SMARCA5 in HMLE cells affected cell motility (**Figure S6C** and **S6D**). To determine the loss-of-function effect, we used a miR-Zip method to achieve lentiviral inhibition of miR-100 in MDA-MB-231 breast cancer cells. Compared with cells infected with a scrambled hairpin control (Zip-scr), cells with approximately 60% knockdown of miR-100 (Zip-100, **Figure 5D**) displayed a significant increase in their migratory and invasive capacity (**Figure 5E**), while their mesenchymal status was not altered (data not shown).

We further validated the effect on tumor invasion *in vivo*: tumors formed by miR-100-overexpressing HMLE-Erbb2 cells were well demarcated and did not show overt invasion to their surrounding tissues (**Figure 5F**); in contrast, tumors formed by either the control HMLE-Erbb2 cells (mock) or HMLE-Erbb2 cells with simultaneous expression of miR-100 and HOXA1 were invasive and infiltrated muscular, adipose and stromal tissues (**Figure 5F**). We conclude from these experiments that miR-100 suppresses migration and invasion, at least in part, through direct targeting of *HOXA1* but not *SMARCA5*.

### miR-100 downregulates HOXA1 downstream targets

*HOXA1* is required for the development of the hindbrain, inner ear and neural crest in mammals [24]–[26]. Genome-wide expression profiling analysis of *Hoxa1*-null mouse embryos identified a number of Hoxa1 downstream targets involved in developmental processes [26]; three of the genes downregulated in *Hoxa1* null embryos, *Met*, *Smo* (smoothened) and *Sema3c* (semaphorin 3c), are positive regulators of tumor cell migration, invasion and/or growth. MET, the receptor for hepatocyte growth factor, has been identified as a driver of tumorigenesis, motility and metastasis [27]. SMO is a central mediator of Hedgehog signaling, whereby Hedgehog binds to the twelve-pass transmembrane protein patched, alleviating patched-mediated inhibition of SMO [28]. It has been shown that the SMO inhibitor cyclopamine can lead to regression of medulloblastoma deficient in patched [29]. SEMA3C is a secreted protein that can induce migratory and invasive properties of breast cancer and prostate cancer cells [30], [31]. In addition, ectopic expression of HOXA1 in MCF7 breast cancer cells upregulated cyclin D1 [20], a cyclin that is required for steroid-induced proliferation of mammary epithelium during pregnancy [32] and promotes the development of mammary adenocarcinomas when overexpressed [33].

**Figure 6:**
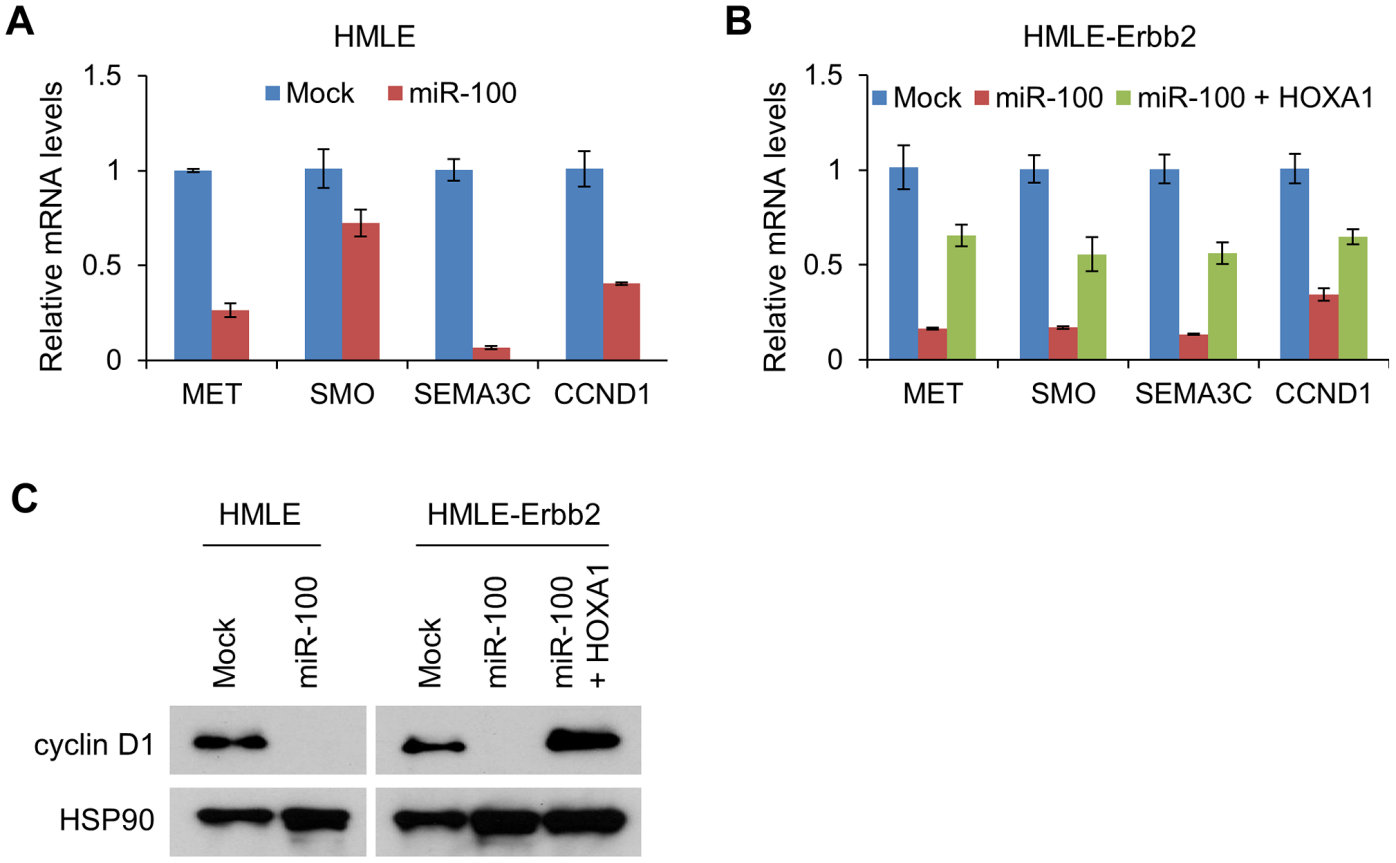
miR-100 downregulates multiple HOXA1 downstream targets involved in tumorigenesis, migration and invasion. (A) qPCR of *MET*, *SMO*, *SEMA3C* and *CCND1* in HMLE cells transduced with miR-100. (B) qPCR of *MET*, *SMO*, *SEMA3C* and *CCND1* in HMLE-Erbb2 cells transduced with the control vector (mock), miR-100 alone or in combination with HOXA1. Data in (A) and (B) are mean ± SEM. (C) Immunoblotting of cyclin D1 and HSP90 in HMLE cells transduced with miR-100, and in HMLE-Erbb2 cells transduced with the control vector (mock), miR-100 alone or in combination with HOXA1.

In the present study, ectopic expression of miR-100 markedly reduced the mRNA levels of *MET*, *SMO*, *SEMA3C* and *CCND1*, either in the presence or absence of Erbb2 expression (**Figure 6A** and **6B**), while restoration of HOXA1 rescued the expression of each of these four genes (**Figure 6B**). A similar effect was observed on cyclin D1 protein expression levels (**Figure 6C**). Therefore, miR-100 downregulates multiple HOXA1 downstream targets involved in oncogenesis and invasiveness.

### miR-100 expression is regulated by ZEB1 and the methylation of the host gene *MIR100HG*

**Figure 7:**
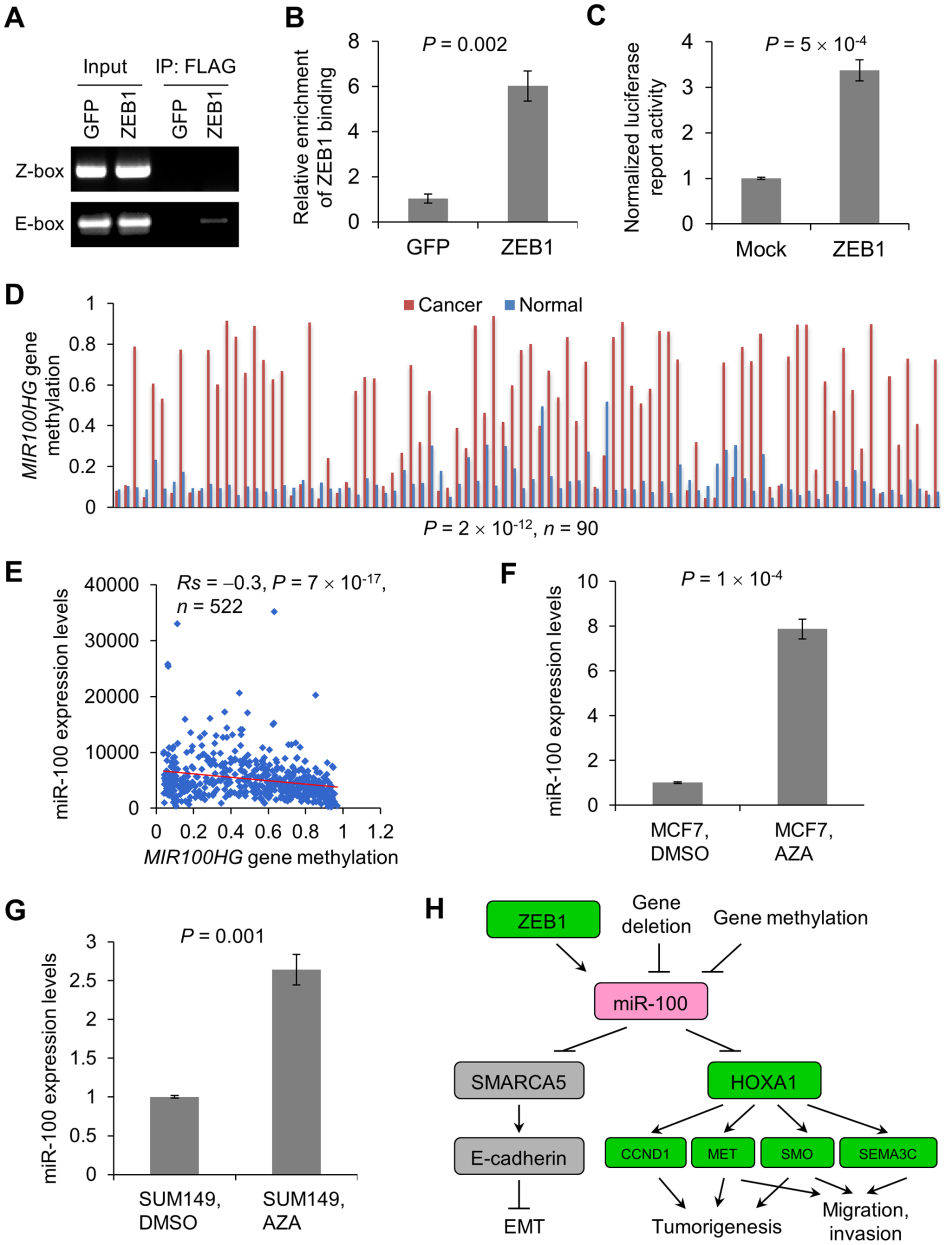
Regulation of miR-100 expression by ZEB1 and the methylation of the host gene *MIR100HG*. (A, B) ChIP-PCR (A) and ChIP-qPCR (B) analysis of ZEB1 binding to the *mir-100* gene in 293T cells transfected with SFB-tagged GFP or ZEB1. PCR was performed with primers specific to the Z-box and E-box elements, respectively. qPCR was performed with primers specific to the E-box element. SFB: S-protein, FLAG tag and streptavidin-binding peptide. (C) Activity of a luciferase reporter fused to the putative human *mir-100* promoter in 293T cells transfected with the control vector (mock) or ZEB1. Data in (B) and (C) are mean ± SEM, and statistical significance was determined by two-tailed, unpaired Student's *t* test. (D) *MIR100HG* gene methylation levels in human breast tumors and paired normal breast tissues, based on the gene methylation data from TCGA. Statistical significance was determined by Wilcoxon signed-rank test. (E) Scattered plot showing the inverse correlation between methylation of the *MIR100HG* gene and miR-100 expression levels in human breast tumors, based on the RNA-Seq data and gene methylation data from TCGA. Statistical significance was determined by Spearman rank correlation test. *Rs* = Spearman rank correlation coefficient. (F, G) qPCR of miR-100 in MCF7 (F) and SUM149 (G) cells treated with the DNA demethylating agent 5-azacytidine (AZA) or vehicle control (DMSO). Data are mean ± SEM, and statistical significance was determined by two-tailed, unpaired Student's *t* test. (H) Model of miR-100-mediated regulation of EMT, tumorigenesis and invasion. Green indicates oncogenic and/or pro-invasive factors; pink indicates tumor-suppressing factors; gray indicates EMT regulators.

We sought to understand how miR-100 expression is regulated. Examination of the 2.5 kb genomic sequence upstream of the human *mir-100* stem-loop identified two putative ZEB1-binding sites at −400 bp (Z-box, CAGGTA) and −2.2 kb (E-box, CAGCTG), respectively (**Figure S7A**). We designed PCR amplicons to assay for the presence of these putative binding sites in chromatin immunoprecipitates. This experiment revealed that ZEB1 bound to the E-box but not to the Z-box (**Figure 7A** and **7B**). Moreover, luciferase assays demonstrated that ZEB1 significantly increased the activity of the putative *mir-100* promoter (**Figure 7C**), suggesting that *mir-100* is likely to be a transcriptional target of ZEB1. Interestingly, overexpression of either Twist or Snail increased ZEB1 expression to the level as high as that of ZEB1-overexpressing cells (**Figure S1B**), which could explain why miR-100 was identified as a commonly upregulated miRNA in HMLE cells overexpressing Twist, Snail or ZEB1. Consistently, miR-100 exhibited a strong positive correlation with Twist (*Rs* = 0.3, *P* = 5×10^−19^), Snail (*Rs* = 0.2, *P* = 4×10^−7^) and ZEB1 (*Rs* = 0.5, *P*<2×10^−16^) expression levels in human breast tumors (**Figure S7B–S7D**)

Upregulation of ZEB1 has been observed in triple-negative and basal-like breast tumors [34], [35]. Paradoxically, miR-100 is commonly downregulated in all subtypes of human breast cancers (**Figure 2A**), which indicates that other mechanisms lead to downregulation of miR-100. The *mir-100* gene is embedded in a non-coding host gene, *MIR100HG*. Analysis of TCGA data revealed that 1.2% of the breast tumors (11 out of a total of 913 samples with copy number data available) had homozygous deletion of both *mir-100* and *MIR100HG*, which could explain loss of miR-100 in these samples. Besides genetic alterations, a second common cause of loss of a tumor suppressor is DNA hypermethylation. From TCGA data, the majority of breast tumors (consisting of luminal A, luminal B, basal-like and HER2 subtypes) had much higher levels of *MIR100HG* gene methylation compared with paired normal mammary tissues (*P* = 2×10^−12^, *n* = 90, **Figure 7D**). Moreover, we observed a significant inverse correlation between *MIR100HG* gene methylation and miR-100 expression levels in breast cancer patients (*Rs* = −0.3, *P* = 7×10^−17^, *n* = 522, **Figure 7E**). Consistently, treatment of MCF7 and SUM149 human breast cancer cell lines with the DNA demethylating agent 5-azacytidine led to significant upregulation of miR-100 expression (**Figure 7F** and **7G**). Taken together, these data suggest that miR-100 expression is regulated by both transcriptional activation and epigenetic silencing.

## Discussion

In summary, we identified miR-100 as a novel EMT inducer and a tumor suppressor, and validated in human tumors that miR-100 is downregulated in clinical breast cancer and correlates with EMT-associated markers. Notably, our results indicate the following: on one hand, both DNA hypermethylation and genetic deletion could contribute to miR-100 downregulation or loss in all subtypes of human breast tumors independently of EMT. On the other hand, induction of miR-100 may serve as a negative feedback mechanism to counteract the tumor-promoting and pro-invasive effect of EMT-inducing transcription factors. However, these transcription factors also regulate many other genes involved in cancer stemness, invasion and metastasis; for example, ZEB1 represses miR-200 [7] and Twist transactivates miR-10b [36]. This appears to be similar to other pleiotropically acting transcription factors: for instance, MYC is a cancer-driving oncoprotein and it is known to transcriptionally activate both pro-survival and pro-apoptotic genes [37].

Because induction of the EMT program can generate stem-like cells [13], [14], we examined the ability of miR-100 to regulate stem cell properties, as gauged by the stem cell marker ALDH1 (aldehyde dehydrogenase 1) [38] and mammosphere-forming ability [39]. Indeed, we observed induction of both ALDH1 expression (**Figure S8A**) and mammosphere formation (**Figure S8B** and **S8C**) by miR-100 in HMLE and HMLE-Erbb2 cells. Thus, the anti-tumor function of miR-100 is not due to depletion of the stem-like cell population, but instead results from inhibition of cell proliferation. In support of this notion, miR-100-expressing HMLE-Erbb2 tumors displayed a 90% reduction in the percentage of Ki-67-positive cells compared with the control HMLE-Erbb2 tumors (**Figure 4E**).

Our work is consistent with the anti-proliferative function of miR-100 described in several recent studies [18], [40], and is the first report of an EMT inducer that suppresses cell movement and invasion. Mechanistically, miR-100 induces EMT by targeting *SMARCA5*, an epigenetic regulator of E-cadherin, and inhibits tumorigenesis, migration and invasion by targeting *HOXA1*, leading to downregulation of multiple HOXA1 downstream targets involved in oncogenesis and invasiveness, including *CCND1*, *MET*, *SMO* and *SEMA3C* (**Figure 7H**). It should be noted that miR-100 has been reported to target *IGF2* in 4T1 mouse mammary tumor cells [40]; however, IGF2 expression is undetectable in the human mammary epithelial cells (HMLE) used in this study (data not shown), although it is possible that IGF2 mediates the function of miR-100 in cells that express IGF2.

Another EMT-inducing miRNA identified in our study is miR-22. Consistent with our results, a recent report also demonstrated that miR-22 is an EMT inducer [41]. However, in stark contrast to miR-100, miR-22 functions to promote tumorigenesis, invasion and metastasis, ostensibly through direct targeting of the TET family of methylcytosine dioxygenases [41]. Although miR-22 expression showed no significant difference between breast tumors and paired normal mammary tissues based on TCGA data analysis (**Figure S3**), patients with high levels of miR-22 had worse survival rates than patients with low levels of miR-22 [41].

Taken together, these results do not argue that EMT itself suppresses cancer, but instead demonstrate that EMT is not always associated with increased tumorigenesis, migration and invasion, and that all EMT inducers are not equal: while some of them (such as miR-22) can promote tumorigenicity, motility and invasiveness, others (such as miR-100) inhibit these properties owing to their ability to target both EMT-repressing genes and oncogenic/pro-invasive genes (**Figure 7H**). Our findings raise the caution that the validity of using EMT-associated gene products as cancer biomarkers should be carefully assessed.

## Materials and Methods

### Cell culture

The HMLE cell line was from R. A. Weinberg's lab stock and cultured in complete Mammary Epithelial Cell Growth Medium (MEGM from Lonza). The MCF7, T47D, MDA-MB-231 and 293T cell lines were purchased from American Type Culture Collection and were cultured under conditions specified by the manufacturer. The SUM149, SUM159 and SUM229 cell lines were from S. Ethier and cultured as described (http://www.asterand.com/Asterand/human_tissues/149PT.htm). For demethylating studies, the MCF7 and SUM149 cells were treated with 2 µM 5-azacytidine (Sigma) for 12 hours.

### Plasmids and shRNA

The human *mir-100*, *mir-22*, *mir-125b* and *mir-720* genomic sequences were PCR amplified from normal genomic DNA and cloned into the MSCV-PIG or pBabe-puro retroviral vector. A 1.5 kb putative human *mir-100* promoter sequence containing the ZEB1-binding site (E-box) was PCR amplified from normal genomic DNA and cloned into the pGL3-Basic vector. A *HOXA1* 3′ UTR fragment was cloned into the pMIR-REPORT luciferase construct, using the following cloning primers: forward, 5′-ATCTTAGCTGGATATAATGTA-3′; reverse, 5′-TGCTTCATAAATTTCTTCATC-3′. A rat oncogenic (activated) form of Erbb2/NeuNT was from W. Guo. The Twist, Snail and ZEB1 expression constructs were from R. A. Weinberg. The human HOXA1 ORF was from Open Biosystems through MD Anderson's shRNA and ORFeome Core (PLOHS_100003514). The human SMARCA5 shRNA was from Sigma (TRCN0000013215). The human SMARCA5 expression vector was from GeneCopoeia (EX-E2767-Lv105). The miR-Zip construct expressing a short hairpin inhibiting miR-100 was from System Biosciences. The *HOXA1* 3′ UTR mutant was generated using a QuikChange Site-Directed Mutagenesis Kit (Stratagene). The vectors used in this study are listed in **Table S3**.

### RNA isolation and real-time RT-PCR

Total RNA, inclusive of small RNAs, was isolated using the mirVana miRNA Isolation Kit (Ambion) and was then reverse transcribed with an iScript cDNA Synthesis Kit (Bio-Rad). The resulting cDNA was used for qPCR using the TaqMan Gene Expression Assays (Applied Biosystems), and data were normalized to an endogenous control β-actin. Quantification of the mature form of miRNAs was performed using the TaqMan MicroRNA Assay Kit (Applied Biosystems) according to the manufacturer's instructions, and the U6 small nuclear RNA was used as an internal control. Real-time PCR and data collection were performed on a CFX96 instrument (Bio-Rad).

### Lentiviral and retroviral transduction

The production of lentivirus and amphotropic retrovirus and infection of target cells were performed as described previously [42].

### miRNA target analysis

Genes that contain the miR-100-binding site(s) in the 3′ UTR were obtained using the TargetScan program [43] (www.targetscan.org; version 6.2). The RNAhybrid program [44] was used to predict duplex formation between miR-100 and human *HOXA1* 3′ UTR.

### Cell proliferation assay

To determine growth curves, we plated equal numbers of cells in 6-cm dishes. Starting from the next day, cells were trypsinized and counted every day. Cell counts were obtained from a TC10 Automated Cell Counter (Bio-Rad).

### Migration and invasion assays

Transwell migration and Matrigel invasion assays were performed as described previously [36].

### Mammosphere assay

Mammosphere assay was performed according to the vendor (Stemcell Technologies)'s protocol. Briefly, single cell suspensions were seeded in the 6-well ultra-low attachment plate (Corning, 3471) at a density of 3.5–4.0×10^4^ cells in 2 ml of freshly prepared Complete MammoCult Medium (Stemcell Technologies, 05620) per well. After incubation for 7 days, the number of mammospheres that were larger than 40 µm in diameter was counted.

### Luciferase reporter assay

Dual luciferase reporter assays were performed as described previously [36].

### Immunoblotting

Western blot analysis was performed with precast gradient gels (Bio-Rad) using standard methods. Briefly, cells were lysed in the RIPA buffer containing protease inhibitors (Roche) and phosphatase inhibitors (Sigma). Proteins were separated by SDS-PAGE and blotted onto a nitrocellulose membrane (Bio-Rad). Membranes were probed with the specific primary antibodies, followed by peroxidase-conjugated secondary antibodies. The bands were visualized by chemiluminescence (Denville Scientific). The following antibodies were used: antibodies to E-cadherin (1∶1000, BD Transduction Laboratories, 610182), vimentin (1∶2000, NeoMarkers, MS-129-P), Erbb2 (1∶500, Cell signaling Technology, 2242), HOXA1 (1∶1000, Santa Cruz Biotechnology, sc-17146), SMARCA5 (1∶500, sc-8760 from Santa Cruz Biotechnology and ab3749 from Abcam), SMARCD1 (1∶500, Abcam, ab86029), mTOR (1∶1000, Cell signaling Technology, 2972), BMPR2 (1∶1000, Cell signaling Technology, 69679), cyclin D1 (1∶1000, Cell signaling Technology, 2922), ALDH1A1 (1∶1000, Santa Cruz Biotechnology, sc-22589), HSP90 (1∶3000, BD Transduction Laboratories, 610419) and cyclophilin B (1∶2000, Thermo, PA1-027A).

### Chromatin immunoprecipitation (ChIP) assay

ChIP was performed with 293T cells transfected with SFB-tagged GFP or ZEB1, by using a Chromatin Immunoprecipitation (ChIP) Assay Kit (Millipore, 17–295) according to the manufacturer's instructions. After immunoprecipitation with FLAG antibody-conjugated beads (Sigma, M8823), protein-DNA crosslinks were reversed and DNA was purified to remove the chromatin proteins and used for PCR and qPCR. The PCR primers are: E-box, 5′-TACTAGGTCAGTATTTGATTT-3′ (forward) and 5′-GTTAGCGATAGACTAAGATCTAT-3′ (reverse); Z-box, 5′-ACCTATAAATCCGTTGGTAG-3′ (forward) and 5′-AATCTGGGCAAAGTGATACC-3′ (reverse). The qPCR primers are 5′-ACTTTGGATTGTTTGGAGGTTAAC-3′ (forward) and 5′-AATTTGCATGGCGCTCTTG-3′ (reverse).

### Bisulfite sequencing

Genomic DNA was extracted using the DNeasy Kit (Qiagen, 69504). The MethylDetector kit (Active Motif, 55001) was used to generate bisulfite-modified DNA. The modified DNA was purified and used as the template for nested PCR reactions with the following primers: outer primers, 5′-ATTCGAATTTAGTGGAATTAGAATC-3′ (forward) and 5′-AACCTACAACAACAACAACAACG-3′ (reverse); nested primers, 5′-TTAGTAATTTTAGGTTAGAGGGTTATCG-3′ (forward) and 5′-ACTCCAAAAACCCATAACTAACCG-3′ (reverse). The second-round PCR products were subcloned into the TOPO cloning vector (Invitrogen, K4600-01) and clones were randomly picked for DNA sequencing.

### *In situ* hybridization

The double (5′ and 3′) digoxigenin (DIG)-labeled miR-100 probe and U6 probe were purchased from Exiqon. The normal mammary tissue and breast tumor sections were purchased from Origene (normal: CS807851; tumor: CS704488 and CS 711714). The tissue microarray (TMA) slide was purchased from Biomax (BR1006). *In situ* hybridization was performed according to the protocol of the miRCURY LNA microRNA ISH Optimization Kit (FFPE) (Exiqon). The stained slide was scanned on the Automated Cellular Image System III (ACIS III, Dako, Denmark) for quantification by digital image analysis. The color threshold was set up and standardized for all samples, and the color intensity was automatically scored for all individual cores on the TMA slide. The expression level was calculated from the score of color intensity and normalized to the internal control U6.

### Time-lapse video microscopy

The 3.5-cm glass bottom multi-well plates (MatTek Corporation) were covered with 1 ml of 1.7 mg/ml collagen solution. After collagen solidified, we seeded 1×10^5^ cells in serum-free and growth factor-free medium on top of the collagen. The cells were incubated overnight, and then were observed for 24 hours in a humidified, CO2-equilibrated chamber mounted on a Zeiss Axio Observer Z1 microscope. To quantitate the speed, we tracked the distance of individual cell movement by using Axio Vision software (Zeiss) in randomly selected fields. The speed of movement was calculated and presented as micrometers per minute.

### miRNA profiling analysis

Agilent human miRNA 8×15K microarray was used to profile global miRNA expression with standard procedures. Arrays were scanned using an Agilent scanner and data were extracted using Agilent's Feature Extraction software set to the default miRNA analysis protocol. The raw data were normalized and quantified by the LIMMA (linear models for microarray data) library, part of the Bioconductor project, using the R statistical environment. The raw data from all arrays were first background-corrected and then normalized using quantile normalization. The difference in miRNA expression between different groups was analyzed using empirical Bayes method implemented in the LIMMA package. *P* values obtained from the multiple comparison tests were corrected by false discovery rates.

### Animal study

Six- to eight-week-old athymic female nude mice were used for tumor cell implantation. Cells were injected subcutaneously into the left back of recipient animals. For recipients of MCF7 cells, Depo-Estradiol (Pfizer) was given to the mice two days before tumor cell implantation (1.5 mg/kg body weight), and the same dose was given once a week after implantation. Tumor size was measured weekly using a caliper, and tumor volume was calculated using the standard formula: 0.5×L×W^2^, where L is the longest diameter and W is the shortest diameter. Mice were euthanized when they met the institutional euthanasia criteria for tumor size and overall health condition. The tumors were removed and weighed. The harvested tumor samples were fixed in 10% buffered formalin for 12 h, washed with PBS, transferred to 70% ethanol, embedded in paraffin, sectioned and stained with hematoxylin and eosin (H & E).

### Immunohistochemistry

Samples were deparaffinized and rehydrated. Antigen retrieval was done using 0.01 M sodium-citrate buffer (pH 6.0) at a sub-boiling temperature for 10 min after boiling in a microwave oven. To block endogenous peroxidase activity, the sections were incubated with 3% hydrogen peroxide for 10 min. After 1 h of preincubation in 5% normal goat serum to prevent nonspecific staining, the samples were incubated with the antibody to Ki-67 (1∶50, BD Biosciences, 550609) or E-cadherin (1∶500, Cell signaling Technology, 3195) at 4°C overnight. The sections were incubated with a biotinylated secondary antibody (1∶500, Vector Laboratories, BA-9200) and then incubated with avidin-biotin peroxidase complex solution (Vector Laboratories, PK-6100) for 30 min at room temperature. Color was developed using the Diaminobenzidine (DAB) substrate kit (BD Biosciences, 550880). Counterstaining was carried out using Harris modified hematoxylin.

### TCGA data analysis

We obtained level 3 data of mRNA expression, miRNA expression and gene methylation of human breast tumors from Synapse (http://synapse.org) (syn1461151). The mRNA expression levels (RNA-Seq by Expectation Maximization, RSEM) were measured by Illumina HiSeq (V2). The miRNA expression levels (normalized read counts) were measured by Illumina HiSeq and Illumina Genome Analyzer. The DNA methylation level (β value) was measured by the Illumina Infinium Human DNA Methylation 450 platform. The breast cancer subtype information (luminal A, luminal B, basal-like and HER2 subtypes) was described previously [17]. Paired *t* test was used to compare miRNA expression levels in all cases with miRNA expression data available from paired normal and cancer tissues (*n* = 56). Wilcoxon signed-rank test was used to compare *MIR100HG* methylation levels in all cases with gene methylation data available from paired normal and cancer tissues (*n* = 90). Spearman rank correlation test was used to assess the correlation between miR-100 expression level and *MIR100HG* gene methylation level (*n* = 522), and the correlation between miRNA expression levels and mRNA expression levels in all breast cancer samples with both miRNA and mRNA expression data available (*n* = 777).

### Statistical analysis

Each experiment was repeated three times or more. Unless otherwise noted, data are presented as mean ± s.e.m., and two-tailed Student's *t* test was used to compare two groups for independent samples. Statistical methods used for TCGA data analysis are described above. *P*<0.05 was considered statistically significant.

### Ethics statement

All animal experiments were performed in accordance with a protocol approved by the Institutional Animal Care and Use Committee of MD Anderson Cancer Center.

## Supporting Information

Figure S1 Induction of EMT by Twist, Snail or ZEB1. (A) Phase contrast images of HMLE cells transduced with Twist, Snail or ZEB1. (B) mRNA levels of *CDH1*, *CDH2*, *VIM*, *TWIST1*, *SNAI1* and *ZEB1* in HMLE cells transduced with Twist, Snail or ZEB1, as gauged by qPCR. Data are mean ± SEM.
(TIF)

Figure S2 Expression levels of miR-100 and miR-22. (A) Heat map showing expression levels of the 13 EMT-associated miRNAs identified by miRNA microarray profiling analysis. (B) qPCR of miR-100 and miR-22 in HMLE cells transduced with miR-100 or miR-22, respectively. (C) qPCR of miR-100 in MCF7 cells transduced with miR-100. Data in (A) and (B) are mean ± SEM.
(TIF)

Figure S3 TCGA data analysis of miR-22. miR-22 expression levels in four subtypes of human breast tumors and paired normal breast tissues. Statistical significance was determined by paired *t* test.
(TIF)

Figure S4 miR-100 inhibits cell proliferation and induces EMT. (A) Growth curves of mock-infected and miR-100-expressing HMLE cells in the absence (left panel) or presence (right panel) of Erbb2 overexpression. Data are mean ± SEM, and statistical significance was determined by two-tailed, unpaired Student's *t* test. (B) E-cadherin immunohistochemical staining of the tumors formed by mock-infected or miR-100-transduced HMLE-Erbb2 cells. Scale bar: 100 µm.
(TIF)

Figure S5 Examination of different miR-100 targets. (A) Immunoblotting of SMARCD1, mTOR, BMPR2 and HSP90 in HMLE cells transduced with miR-100. (B) Upper panel: duplex formation between human *HOXA1* 3′ UTR (the sequence in red) and miR-100 (the sequence in green) as predicted by the RNAhybrid program. Lower panel: sequence of the miR-100 binding site within the *HOXA1* 3′ UTR of human (hs) and mouse (mm); a mutant 3′ UTR of human *HOXA1* containing mutations in the miR-100 binding site (mut) was used for luciferase reporter assays in Figure 3B. (C) Growth curves of HMLE cells infected with the *SMARCA5* shRNA (shSMARCA5) or the pLKO.1-puro lentiviral vector with a scrambled sequence. Data are mean ± SEM, and statistical significance was determined by two-tailed, unpaired Student's *t* test. (D) Immunoblotting of E-cadherin, vimentin and cyclophilin B (CypB) in Erbb2-expressing HMLE (HMLE-Erbb2) cells transduced with the control vector (mock), miR-100 alone or in combination with HOXA1.
(TIF)

Figure S6 miR-100, but not SMARCA5, inhibits cell migration and invasion. (A) Representative images of Transwell migration and Matrigel invasion assays of HMLE-Erbb2 cells transduced with the control vector (mock), miR-100 alone or in combination with HOXA1. (B) Representative images of Transwell migration and Matrigel invasion assays of miR-100-transduced MCF7 cells. (C, D) Transwell migration assays of HMLE cells transduced with the control vector (mock), miR-100 alone or in combination with HOXA1 (C), and of SMARCA5 shRNA-transduced HMLE cells (D). Data are mean ± SEM, and statistical significance was determined by two-tailed, unpaired Student's *t* test.
(TIF)

Figure S7 miR-100 correlates with Twist, Snail and ZEB1 expression levels in human breast tumors. (A) Schematic representation of human *mir-100* genomic locus. The two short blue lines represent PCR amplicons specific to the Z-box and E-box elements, respectively. (B–D) Correlation of miR-100 with *TWIST1* (B), *SNAI1* (C) and ZEB1 (D) expression levels in clinical breast cancer, based on the RNA-Seq data from TCGA. Statistical significance was determined by Spearman rank correlation test. *Rs* = Spearman rank correlation coefficient.
(TIF)

Figure S8 miR-100 increases stem-like cell population. (A) Immunoblotting of ALDH1 and HSP90 in miR-100-transduced HMLE and HMLE-Erbb2 cells. (B, C) Quantification (B) and images (C) of mammosphere formation by miR-100-transduced HMLE and HMLE-Erbb2 cells. Data in (B) are mean ± SEM, and statistical significance was determined by two-tailed, unpaired Student's *t* test.
(TIF)

Table S1 miRNA micaroarray analysis of HMLE cells expressing Twist, Snail or ZEB1.
(XLSX)

Table S2 Expression levels of EMT-associated miRNAs in HMLE cells expressing Twist, Snail or ZEB1. Data from miRNA microarray analysis and TaqMan qPCR validation are normalized to the control (mock-infected) HMLE cells.
(XLSX)

Table S3 Vectors used in this study.
(XLSX)

Video S1 Time-lapse video microscopy of HMLE-Erbb2 cells. An epithelial cell cluster initially appeared in the upper left corner and then moved to the lower part of the field.
(MPG)

Video S2 Time-lapse video microscopy of miR-100-transduced HMLE-Erbb2 cells.
(MPG)

Video S3 Time-lapse video microscopy of HMLE-Erbb2 cells transduced with both miR-100 and HOXA1.
(MPG)
