# Supplementary figures and images for: miR-100 Induces Epithelial-Mesenchymal Transition but Suppresses Tumorigenesis, Migration and Invasion
Source: PLoS Genet. 2014 Feb 27;10(2):e1004177. doi: 10.1371/journal.pgen.1004177 (PMC3937226; doi:10.1371/journal.pgen.1004177)

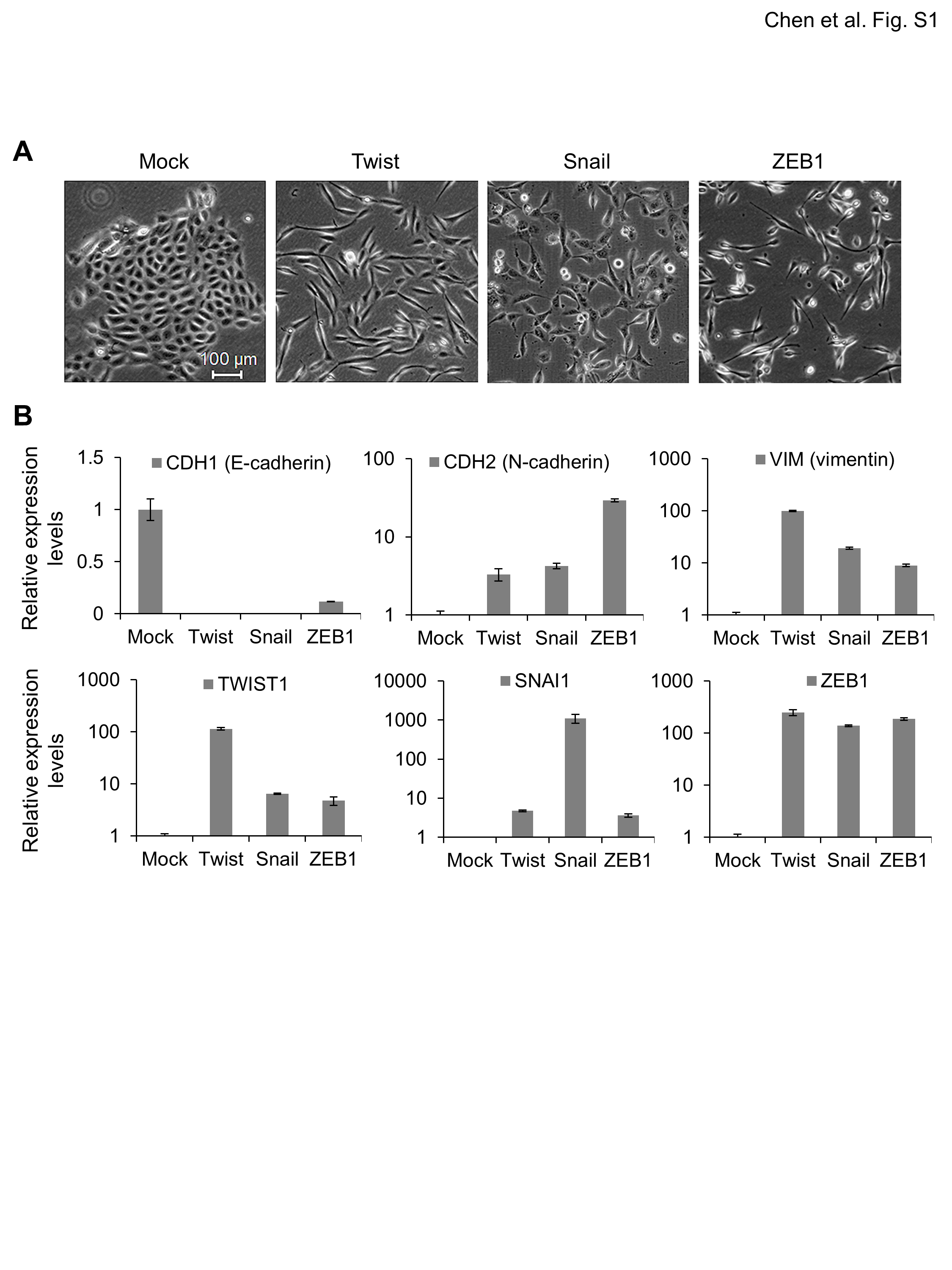

Supplement: Figure S1 — Induction of EMT by Twist, Snail or ZEB1. (A) Phase contrast images of HMLE cells transduced with Twist, Snail or ZEB1. (B) mRNA levels of CDH1, CDH2, VIM, TWIST1, SNAI1 and ZEB1 in HMLE cells transduced with Twist, Snail or ZEB1, as gauged by qPCR. Data are mean ± SEM. (TIF) [file pgen.1004177.s001.tif]

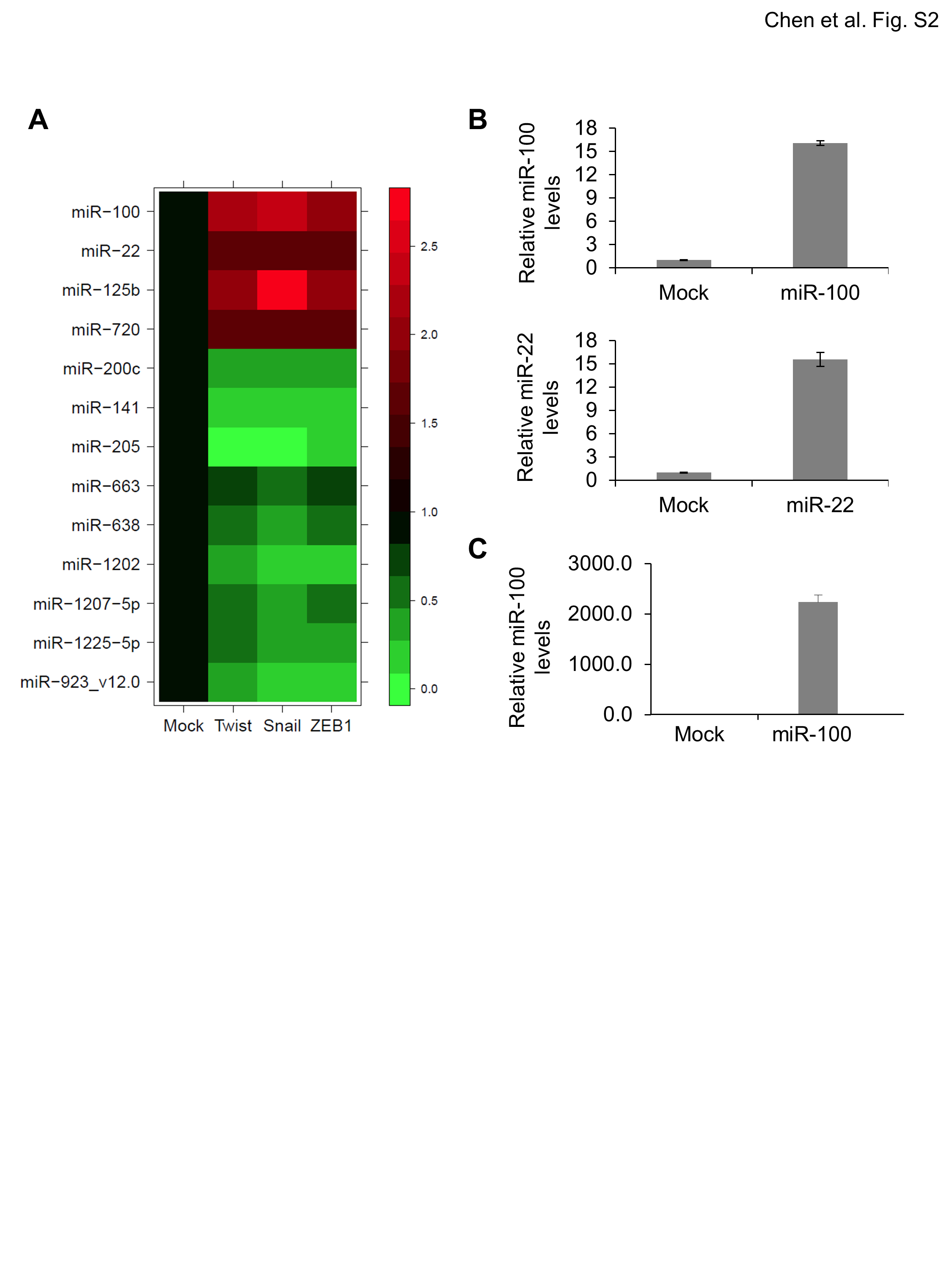

Supplement: Figure S2 — Expression levels of miR-100 and miR-22. (A) Heat map showing expression levels of the 13 EMT-associated miRNAs identified by miRNA microarray profiling analysis. (B) qPCR of miR-100 and miR-22 in HMLE cells transduced with miR-100 or miR-22, respectively. (C) qPCR of miR-100 in MCF7 cells transduced with miR-100. Data in (A) and (B) are mean ± SEM. (TIF) [file pgen.1004177.s002.tif]

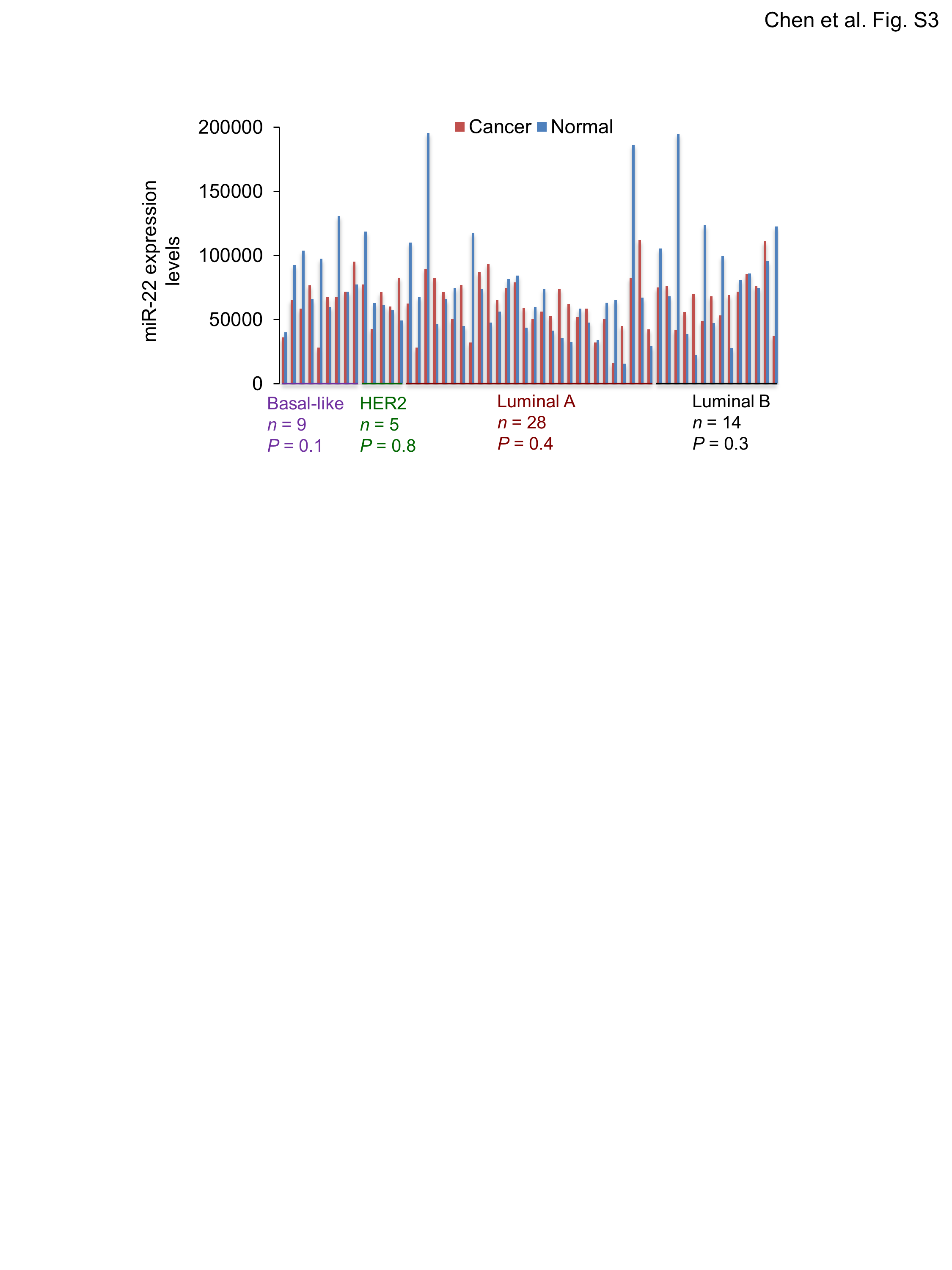

Supplement: Figure S3 — TCGA data analysis of miR-22. miR-22 expression levels in four subtypes of human breast tumors and paired normal breast tissues. Statistical significance was determined by paired t test. (TIF) [file pgen.1004177.s003.tif]

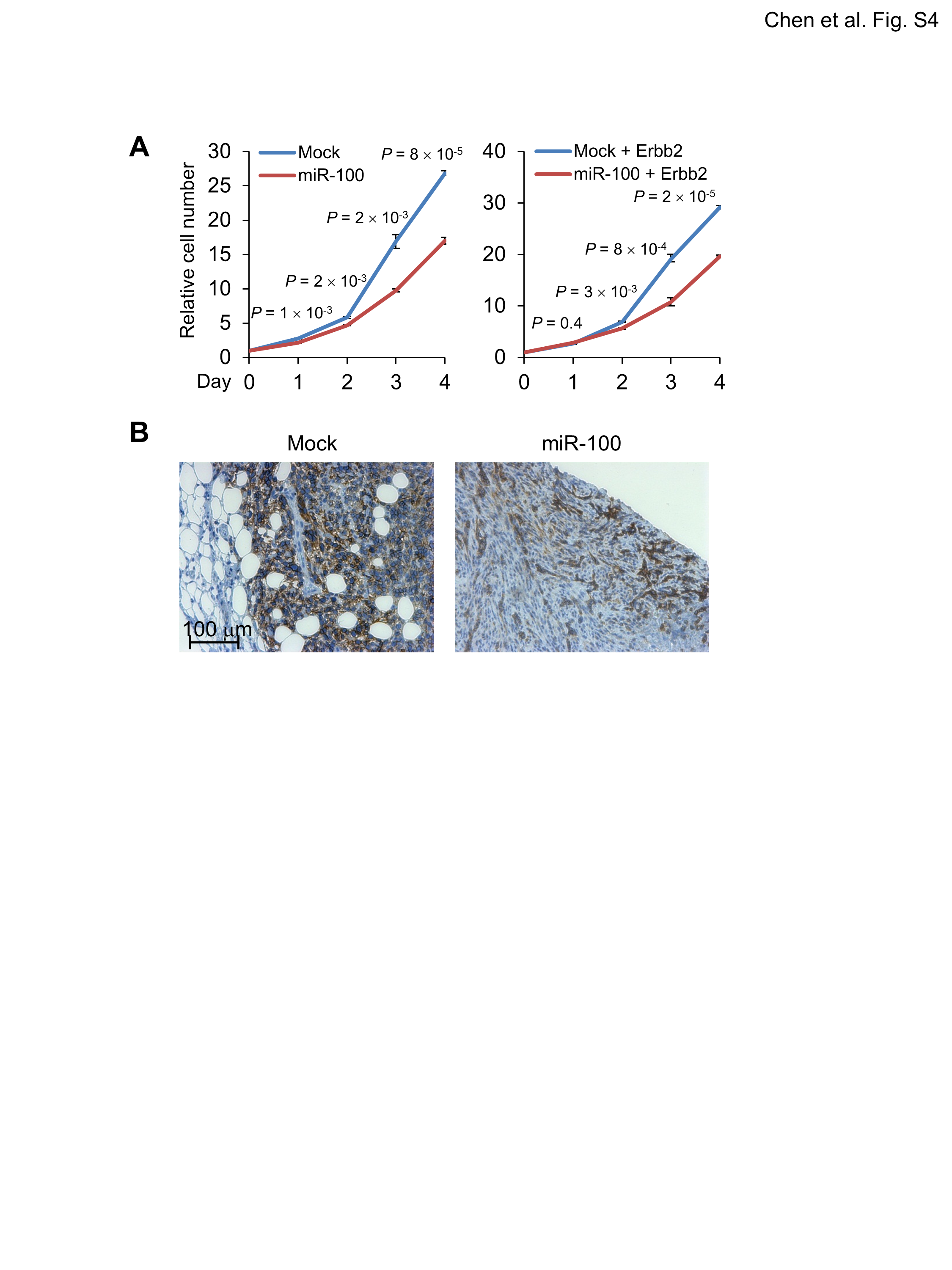

Supplement: Figure S4 — miR-100 inhibits cell proliferation and induces EMT. (A) Growth curves of mock-infected and miR-100-expressing HMLE cells in the absence (left panel) or presence (right panel) of Erbb2 overexpression. Data are mean ± SEM, and statistical significance was determined by two-tailed, unpaired Student's t test. (B) E-cadherin immunohistochemical staining of the tumors formed by mock-infected or miR-100-transduced HMLE-Erbb2 cells. Scale bar: 100 µm. (TIF) [file pgen.1004177.s004.tif]

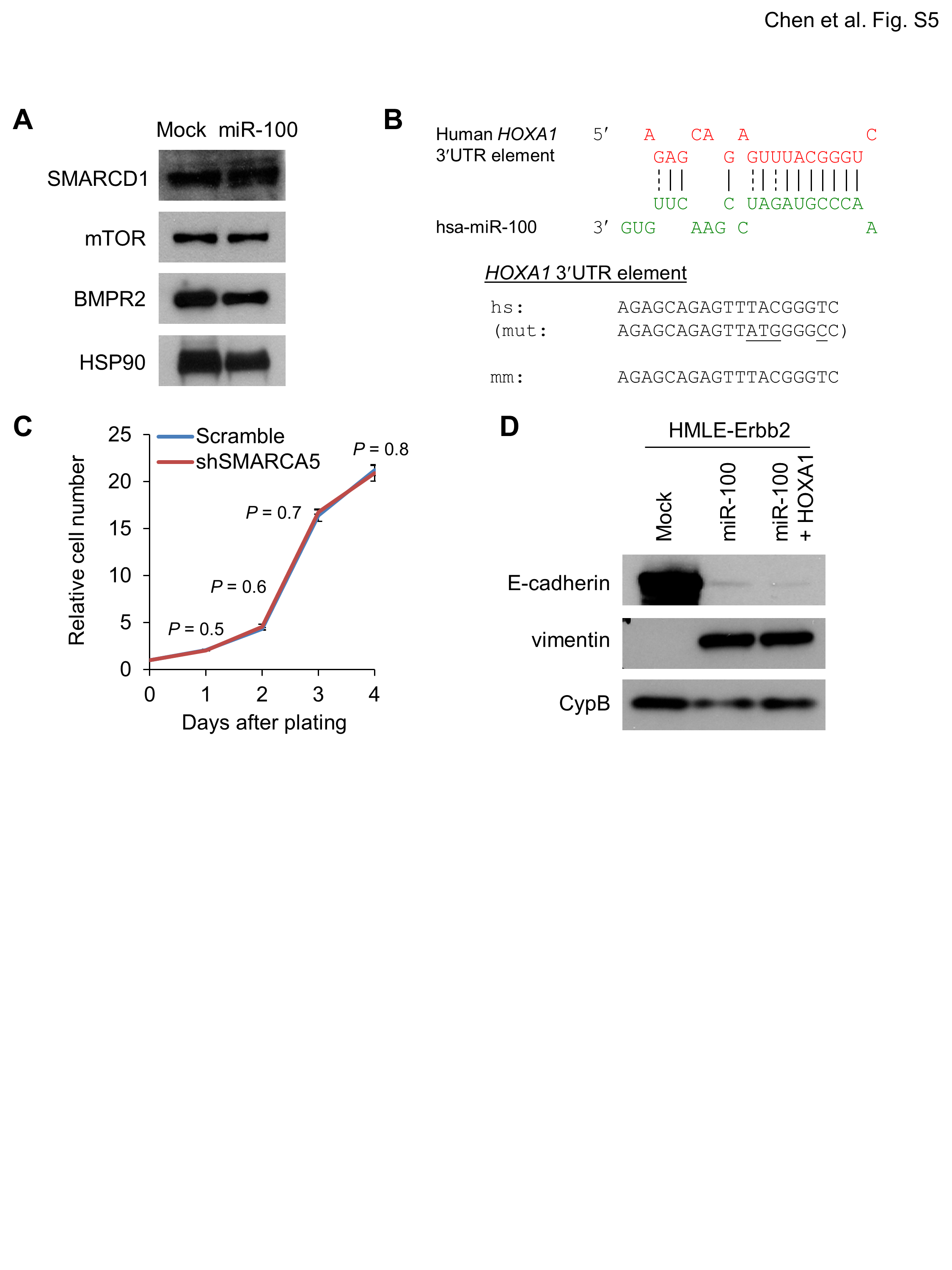

Supplement: Figure S5 — Examination of different miR-100 targets. (A) Immunoblotting of SMARCD1, mTOR, BMPR2 and HSP90 in HMLE cells transduced with miR-100. (B) Upper panel: duplex formation between human HOXA1 3′ UTR (the sequence in red) and miR-100 (the sequence in green) as predicted by the RNAhybrid program. Lower panel: sequence of the miR-100 binding site within the HOXA1 3′ UTR of human (hs) and mouse (mm); a mutant 3′ UTR of human HOXA1 containing mutations in the miR-100 binding site (mut) was used for luciferase reporter assays in Figure 3B. (C) Growth curves of HMLE cells infected with the SMARCA5 shRNA (shSMARCA5) or the pLKO.1-puro lentiviral vector with a scrambled sequence. Data are mean ± SEM, and statistical significance was determined by two-tailed, unpaired Student's t test. (D) Immunoblotting of E-cadherin, vimentin and cyclophilin B (CypB) in Erbb2-expressing HMLE (HMLE-Erbb2) cells transduced with the control vector (mock), miR-100 alone or in combination with HOXA1. (TIF) [file pgen.1004177.s005.tif]

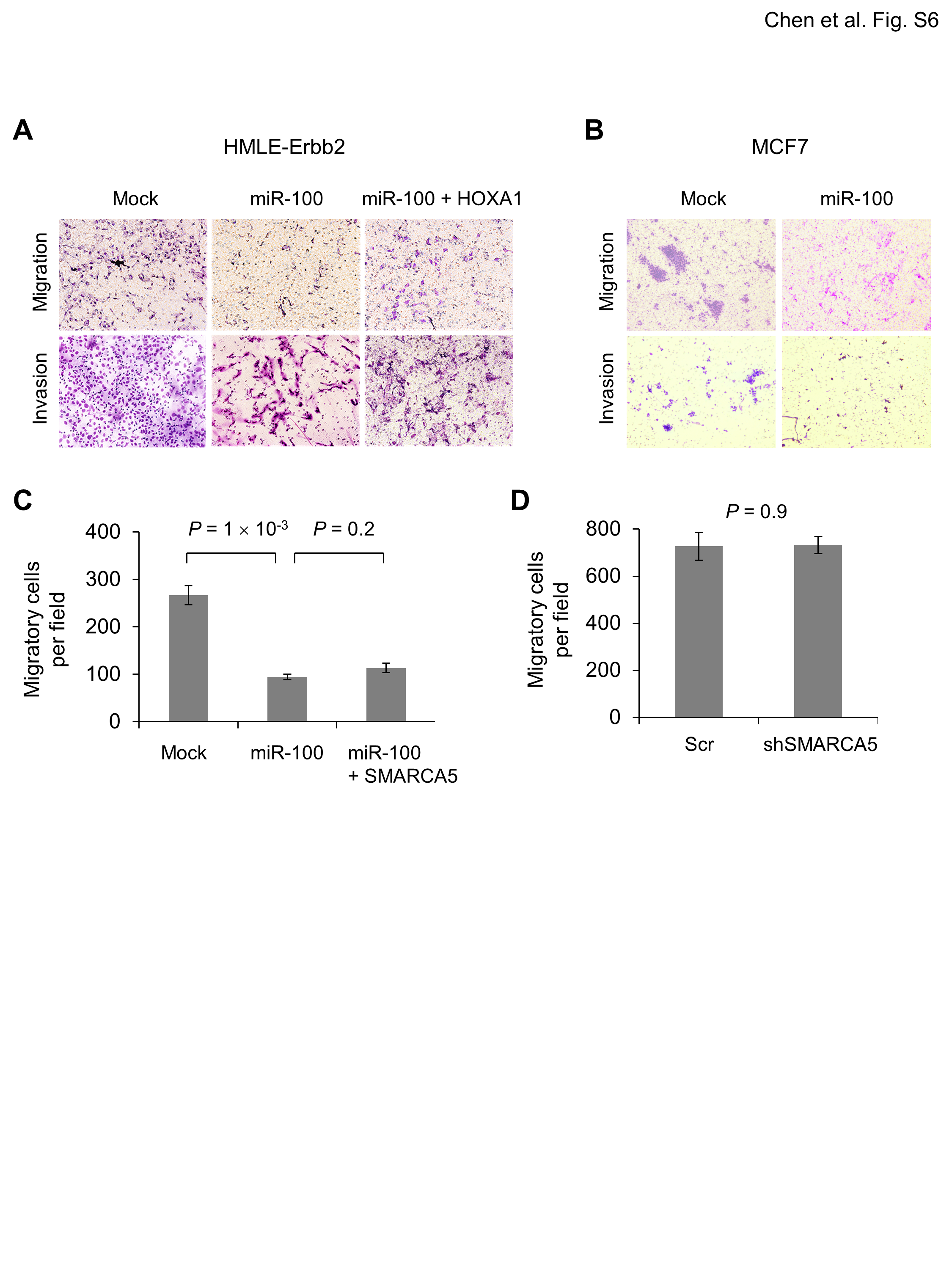

Supplement: Figure S6 — miR-100, but not SMARCA5, inhibits cell migration and invasion. (A) Representative images of Transwell migration and Matrigel invasion assays of HMLE-Erbb2 cells transduced with the control vector (mock), miR-100 alone or in combination with HOXA1. (B) Representative images of Transwell migration and Matrigel invasion assays of miR-100-transduced MCF7 cells. (C, D) Transwell migration assays of HMLE cells transduced with the control vector (mock), miR-100 alone or in combination with HOXA1 (C), and of SMARCA5 shRNA-transduced HMLE cells (D). Data are mean ± SEM, and statistical significance was determined by two-tailed, unpaired Student's t test. (TIF) [file pgen.1004177.s006.tif]

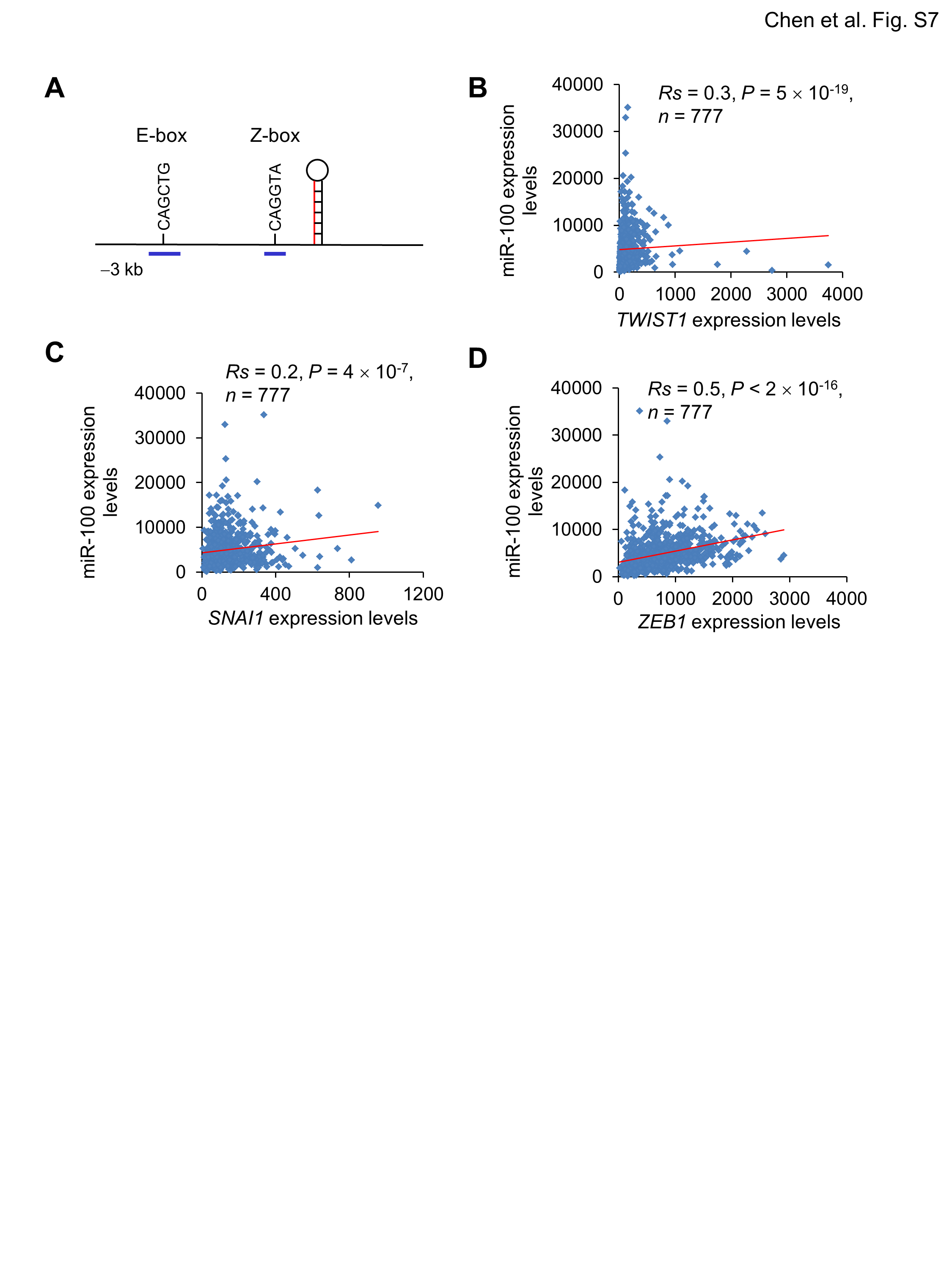

Supplement: Figure S7 — miR-100 correlates with Twist, Snail and ZEB1 expression levels in human breast tumors. (A) Schematic representation of human mir-100 genomic locus. The two short blue lines represent PCR amplicons specific to the Z-box and E-box elements, respectively. (B–D) Correlation of miR-100 with TWIST1 (B), SNAI1 (C) and ZEB1 (D) expression levels in clinical breast cancer, based on the RNA-Seq data from TCGA. Statistical significance was determined by Spearman rank correlation test. Rs = Spearman rank correlation coefficient. (TIF) [file pgen.1004177.s007.tif]
